# Mahaim pathway potential revealed by high-resolution three-dimensional mapping

**DOI:** 10.1007/s00399-020-00721-8

**Published:** 2020-09-28

**Authors:** Johannes Steinfurt, Christoph Bode, Thomas S. Faber

**Affiliations:** Department of Cardiology and Angiology, University Heart Center Freiburg—Bad Krozingen, Faculty of Medicine, University of Freiburg, Hugstetter Str. 55, 79106 Freiburg, Germany

**Keywords:** 3D mapping, Multi-electrode catheter, Atriofascicular pathway, Mahaim fiber, Catheter ablation, 3D-Mapping, Multi-Elektroden-Katheter, Atriofaszikuläre Leitungsbahn, Mahaim-Bündel, Katheterablation

## Abstract

Mapping and ablation of atriofascicular fibers can be highly challenging due to the complex and dynamic anatomy of the tricuspid valve annulus. This case highlights the utility of a multi-electrode catheter three-dimensional mapping approach to localize the Mahaim pathway along the tricuspid annulus in order to guide catheter ablation.

## Abstract (de)

Das Mapping und die Katheterablation atriofaszikulärer Fasern wird durch die komplexe und dynamische Anatomie des Trikuspidalklappenrings erschwert. Der vorliegende Fall zeigt die Vorzüge eines 3‑D-Mapping-Ansatzes mit einem Multi-Elektroden-Mapping-Katheter zur Lokalisation und Ablation des Mahaim-Bündels am Trikuspidalklappenring.

## Case report

**Fig. 1:**
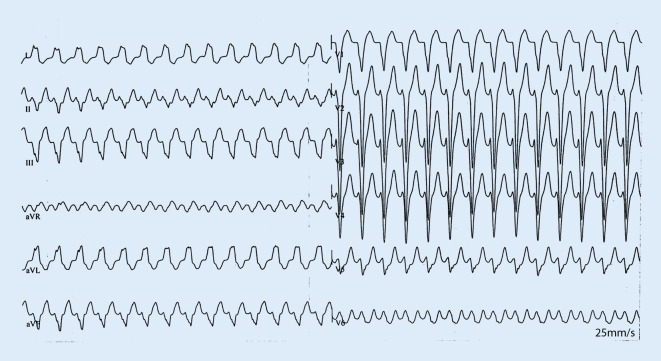
Clinical tachycardia. Wide complex tachycardia at a rate of 170 bpm with left axis deviation, late transition left bundle branch block, and QRS duration of 138 ms indicating an origin at the right ventricular free wall insertion of the moderator band where the right bundle arborizes

**Fig. 2:**
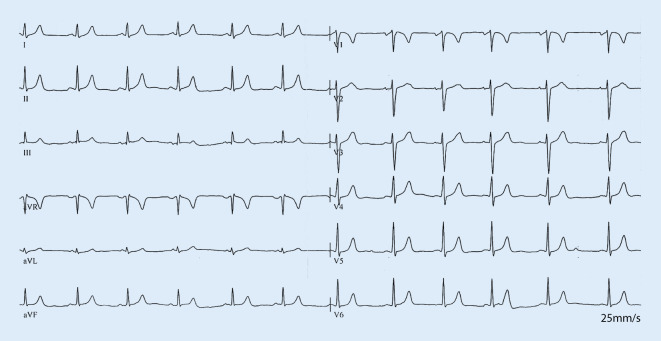
Baseline 12-lead electrocardiogram

A 20-year-old woman was admitted for recurrent wide complex tachycardia (Fig. 1). The baseline electrocardiogram (ECG) showed normal sinus rhythm with a short PR interval of 130 ms, absence of septal Q waves, and no manifest pre-excitation (Fig. 2). Echocardiography was unremarkable.

**Fig. 3:**
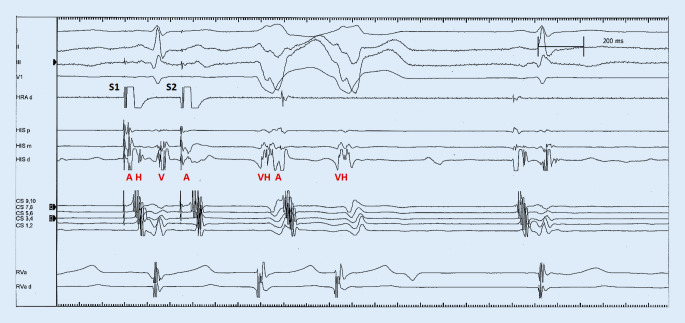
Antidromic echo. The atrial premature beat is conducted antegrade over the accessory pathway followed by retrograde His activation and an antidromic echo beat with retrograde block in the AV node. Note long stim‑V interval, left axis deviation and left bundle branch block pattern in V1 after S2. *HRA* high right atrium, *CS* coronary sinus, *RVa* right ventricular apex

**Fig. 4:**
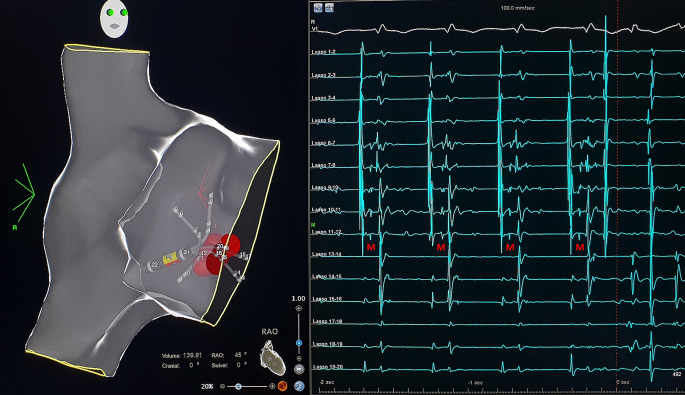
Mahaim potential. Mahaim “M” pathway potential recorded on the inner bipoles (7.8 and 11.12) of the Pentaray® catheter (Biosense Webster, Irvine, CA, USA) facing the lateral TA. Ablation tags indicate sites with Mahaim automaticity during radiofrequency ablation

**Fig. 5:**
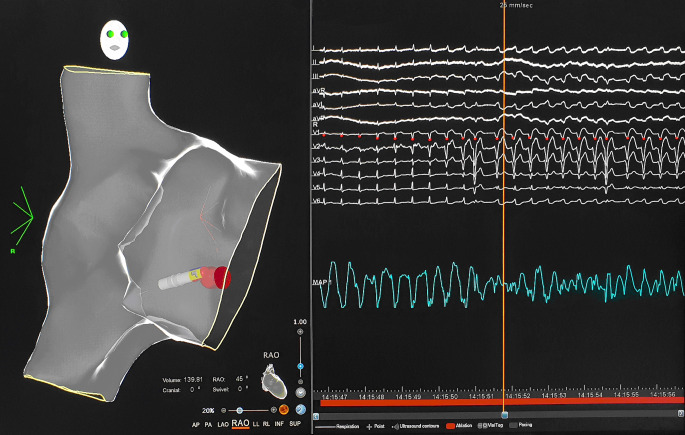
Mahaim automaticity. Mahaim automaticity induced by radiofrequency ablation (30–40 Watts, 30–60 sec., contact force > 10 g) at the lateral TA using an open-tip irrigation contact force catheter (ThermoCool SmartTouch®, Biosense Webster) and an Agilis® sheath (St. Jude Medical/Abbott)

Programmed atrial stimulation resulted in stim-V interval prolongation with increased preexcitation (HV shortening) and induction of antidromic echo beats with concentric atrial activation (Fig. 3) followed by atrioventricular reciprocating tachycardia (AVRT) matching the clinical tachycardia. The tachycardia was reset and terminated by a single ventricular extrastimulus. The morphology (Fig. 1) and the response to pacing maneuvers were compatible with a decremental atriofascicular Mahaim pathway as the antegrade limb of the AVRT circuit [1]. Due to low success rates of conventional, fluoroscopy-guided cases to detect a Mahaim potential [2], a three-dimensional (3D) mapping approach (CARTO® 3, Biosense Webster, Irvine, CA, USA) was chosen. A sharp and consistent “M” potential was recorded at the lateral tricuspid annulus (TA) (Fig. 4) using a multi-electrode Pentaray® catheter (Biosense Webster) with small, tightly spaced electrodes and a steerable Agilis® sheath (St. Jude Medical/Abbott, USA). Radiofrequency ablation at this location elicited Mahaim automaticity (Fig. 5) and the tachycardia became non-inducible. The accessory pathway (AP) potential was absent upon re-mapping and there has been no recurrence during 6 months of follow-up.

A 3D mapping approach may be particularly useful in the case of Mahaim pathways [3] as these endocardial fibers are extremely susceptible to mechanical trauma during catheter manipulation. Direct bumping is common with ablation catheters placed perpendicular to the AP, and the pathway may disappear for hours [1, 3]. Multi-electrode mapping catheters, especially the soft and flexible Pentaray® splines, may be ideally suited to localize the Mahaim pathway potential along the TA sulcus as they combine high-resolution mapping with minimal risk of mechanical AP block. If the pathway has been accidentally blocked, the AP can readily be relocated as the mapping system saves every beat and the corresponding catheter position and electrograms. In fact, using a Pentaray® for Mahaim APs was first reported by colleagues from Heidelberg after a failed conventional approach [4], and the *novel expert consensus on 3D mapping systems for tachycardia* [5] recommends the use of a 3D mapping system for localization and ablation of APs with lower success and higher recurrence rates, such as right-sided APs. In addition to improving mapping and ablation success, high-definition 3D mapping can provide further insights into the Mahaim anatomy and physiology, as elegantly demonstrated by Nishimura et al., who delineated the entire Mahaim pathway activation from TA to right ventricular breakout site [6].
